# Crystal structure of ethyl 6-methyl-2-oxo-4-(3,4,5-tri­meth­oxy­phen­yl)-1,2,3,4-tetra­hydro­pyrimidine-5-carboxyl­ate

**DOI:** 10.1107/S2056989015003576

**Published:** 2015-02-25

**Authors:** J. J. Novina, G. Vasuki, M. Suresh, M. Syed Ali Padusha

**Affiliations:** a Department of Physics, Idhaya College for Women, Kumbakonam-1, India; b Department of Physics, Kunthavai Naachiar Govt. Arts College (W) (Autonomous), Thanjavur-7, India; c PG & Research Department of Chemistry, Jamal Mohamed College (Autonomous), Tiruchirappalli-20, India

**Keywords:** crystal structure, pyrimidine, hydrogen bonds, centrosymmetric dimer

## Abstract

In the title compound, C_17_H_22_N_2_O_6_, the di­hydro­pyrimidine ring adopts a flattened boat conformation. The dihedral angle between the benzene ring and the mean plane of the di­hydro­pyrimidine ring is 75.25 (6)°. In the crystal, mol­ecules are linked *via* pairs of N—H⋯O hydrogen bonds, forming inversion dimers with an *R*_2_^2^(8) ring motif which are linked through N—H⋯O and weak C—H⋯O hydrogen bonds. These, together with π–π ring inter­actions [centroid–centroid distance = 3.7965 (10) Å], give an overall three-dimensional structure.

## Related literature   

For general background and the biological activity of di­hydro­pyrimidino­nes, see: Jawale *et al.* (2011 ▸); Beşoluk *et al.* (2010 ▸); Karade *et al.* (2007 ▸).
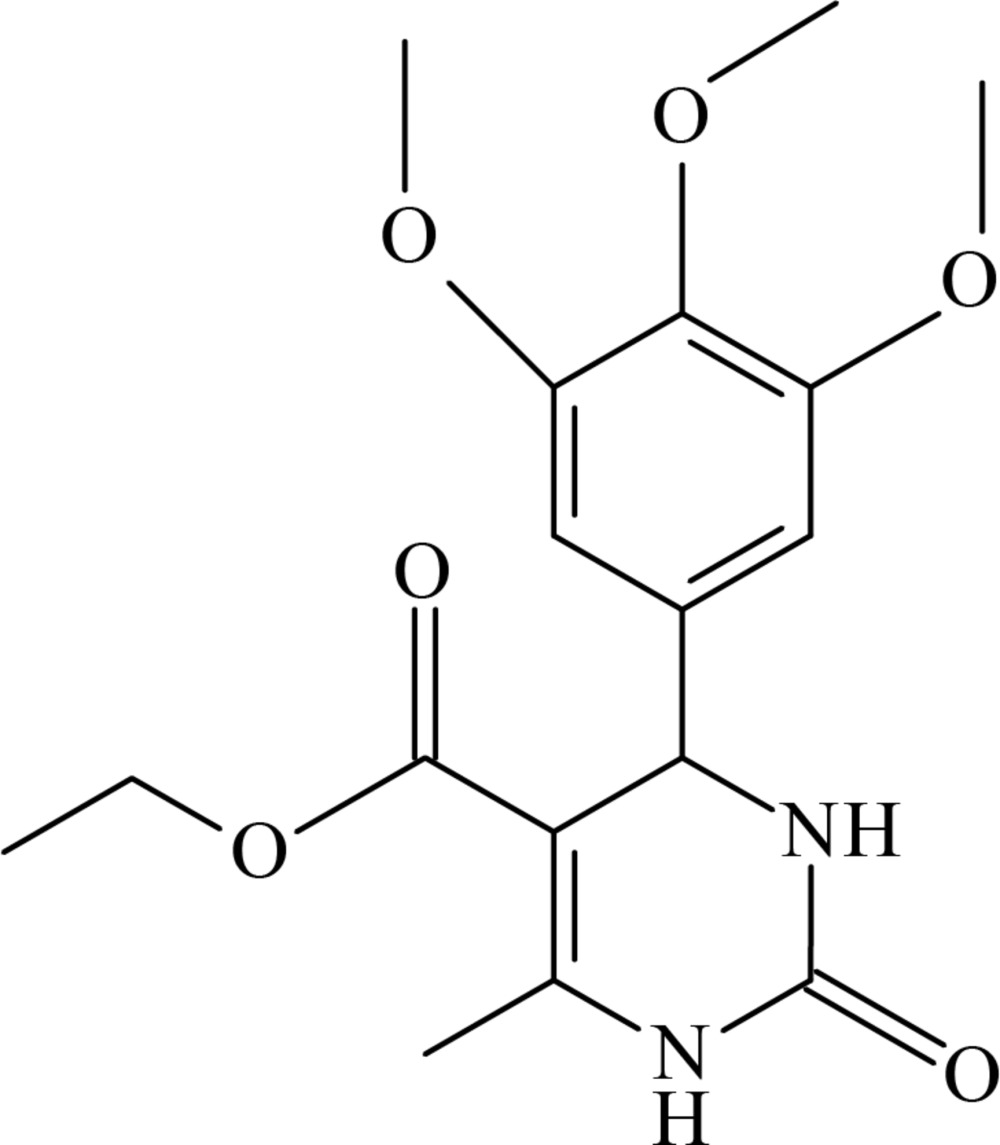


## Experimental   

### Crystal data  

C_17_H_22_N_2_O_6_
*M* *_r_* = 350.37
Triclinic, 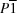

*a* = 10.1447 (3) Å
*b* = 10.1919 (2) Å
*c* = 10.8724 (2) Å
α = 117.882 (1)°
β = 101.371 (1)°
γ = 105.498 (1)°
*V* = 886.40 (4) Å^3^
*Z* = 2
Mo *K*α radiation
μ = 0.10 mm^−1^
*T* = 293 K
0.20 × 0.15 × 0.10 mm

### Data collection  

Bruker Kappa APEXII CCD diffractometer
Absorption correction: multi-scan (*SADABS*; Bruker, 2008 ▸) *T*_min_ = 0.970, *T*_max_ = 0.995
13060 measured reflections
3659 independent reflections
3009 reflections with *I* > 2σ(*I*)
*R*_int_ = 0.020

### Refinement  

*R*[*F* ^2^ > 2σ(*F* ^2^)] = 0.050
*wR*(*F* ^2^) = 0.154
*S* = 1.06
3659 reflections
228 parameters
1 restraint
H-atom parameters constrained
Δρ_max_ = 0.60 e Å^−3^
Δρ_min_ = −0.30 e Å^−3^

### 

Data collection: *APEX2* (Bruker, 2008 ▸); cell refinement: *APEX2* and *SAINT* (Bruker, 2008 ▸); data reduction: *SAINT* and *XPREP* (Bruker, 2008 ▸); program(s) used to solve structure: *SIR92* (Altomare *et al.*, 1993 ▸); program(s) used to refine structure: *SHELXL97* (Sheldrick, 2008 ▸); molecular graphics: *ORTEP-3 for Windows* (Farrugia, 2012 ▸) and *Mercury* (Macrae *et al.*, 2008 ▸); software used to prepare material for publication: *PLATON* (Spek, 2009 ▸).

## Supplementary Material

Crystal structure: contains datablock(s) I, global. DOI: 10.1107/S2056989015003576/zs2327sup1.cif

Structure factors: contains datablock(s) I. DOI: 10.1107/S2056989015003576/zs2327Isup2.hkl

Supporting information file. DOI: 10.1107/S2056989015003576/zs2327Isup3.cml

**Fig. 1.**
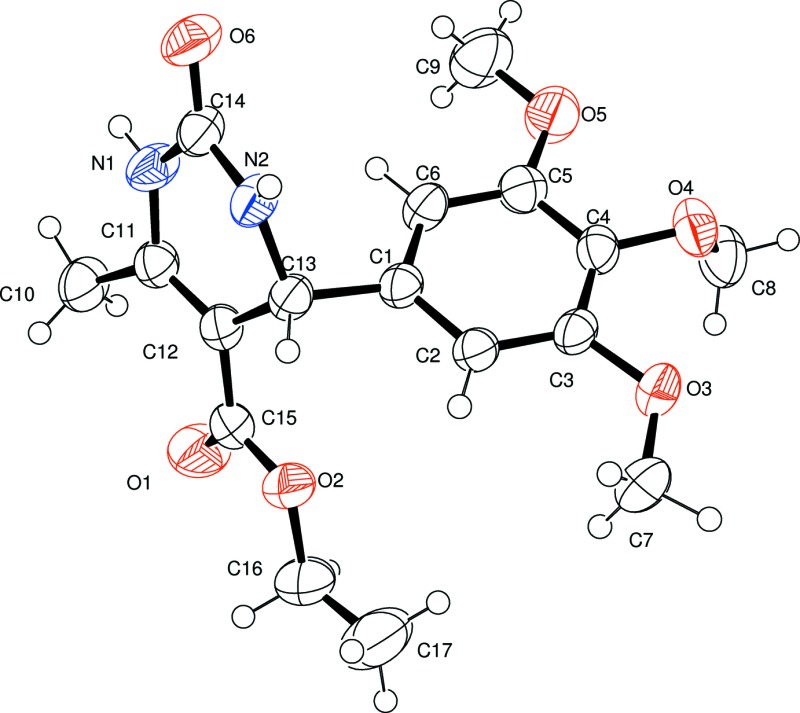
The molecular structure of the title compound, with the atom labelling. Displacement ellipsoids are drawn at the 50% probability level.

**Fig. 2.**
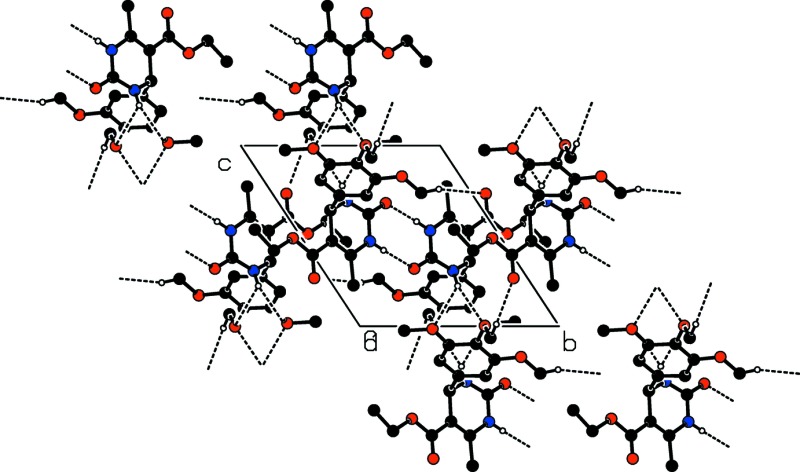
Cystal packing of the title compound viewed along the a axis. Hydrogen bonds are shown as dashed lines (Table 1). For clarity only the H atoms participating in these interactions are shown.

. DOI: 10.1107/S2056989015003576/zs2327fig1.tif
The mol­ecular structure of the title compound, with the atom labelling. Displacement ellipsoids are drawn at the 50% probability level.

a . DOI: 10.1107/S2056989015003576/zs2327fig2.tif
Cystal packing of the title compound viewed along the *a* axis. Hydrogen bonds are shown as dashed lines (Table 1). For clarity only the H atoms participating in these inter­actions are shown.

**Fig. 3.**
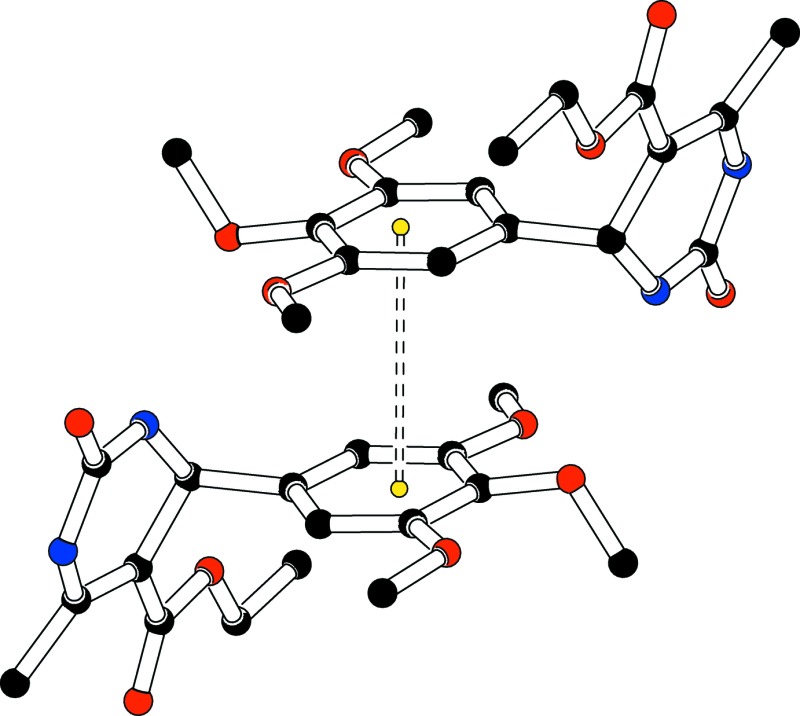
A view showing the π–π interactions. The H atoms are omitted for the sake of clarity.

. DOI: 10.1107/S2056989015003576/zs2327fig3.tif
A view showing the π–π inter­actions. The H atoms are omitted for the sake of clarity.

CCDC reference: 1050728

Additional supporting information:  crystallographic information; 3D view; checkCIF report

## Figures and Tables

**Table 1:** Hydrogen-bond geometry (, )

*D*H*A*	*D*H	H*A*	*D* *A*	*D*H*A*
N1H1*N*O6^i^	0.86	2.01	2.867(2)	171
N2H2*N*O4^ii^	0.86	2.39	3.1331(19)	145
C8H8*A*O1^iii^	0.96	2.46	3.325(3)	149
C9H9*A*O1^iv^	0.96	2.56	3.491(3)	163

Symmetry codes: (i) 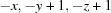; (ii) 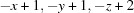; (iii) 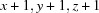; (iv) 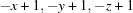.

## supplementary crystallographic information

### S1. Comment

Dihydropyrimidinones (DHPMs) occupy a special place in the areas of natural and synthetic organic chemistry, because of their therapeutic and pharmacological properties. The dihydropyrimidinone scaffold has emerged as an integral backbone for several drugs used as calcium channel blockers as well as anti-hypertensive and anti-cancer agents. DHPMs also exhibit anti-diabetic activity (Jawale *et al.*, 2011). Furthermore, the 2-oxodihydropyrimidine-5-carboxylate core unit is also found in many marine natural products, including the batzelladine alkaloids, which were found to be potent HIV gp-120-CD4 inhibitors (Karade *et al.*, 2007; Beşoluk *et al.*, 2010). Because of this background and in order to obtain detailed information on its molecular conformation, the X-ray structure of the title compound, C_17_H_22_N_2_O_2_ has been determined and is presented herein.

In the racemic title compound (Fig. 1) the dihydropyrimidone ring adopts a flattened boat conformation with the atom C13 and N2 deviating by -0.1218 (11) and 0.1432 (12) Å, respectively from the least squares plane defined by the remaining atoms N1/C11/C12/C14 in the ring. The puckering parameters are q2 = 0.207 Å, q3 = -0.074 Å, Q = 0.220 Å, Θ = 109.7° and Φ = 35.0°. The C1—C6 benzene ring is twisted with respect to the dihydropyrimidinone ring, with an inter-ring dihedral angle of 75.25 (9)°. The ethyl acetate group attached to the pyrimidine ring shows an extended conformation [torsion angle C12—C15—O2—C16 = -177.55 (20)°]. The methoxy two substituent groups at C3 and C5 are almost coplanar with the benzene ring [torsion angles C2—C3—O3—C7 = 7.7 (3)° and C6—C5—O5—C9 = -5.1 (3)°] whereas the central group at C4 deviates significantly from the benzene plane [C3—C4—O4—C8 = 102.7 (2)°].

In the crystal, molecules are linked *via* a pair of N—H···O hydrogen bonds (Table 1) forming a centrosymmetric cyclic dimer with an *R*_2_^2^(8) ring motif. The inter-dimer N2—H···O3^ii^ and N2—H···O4^ii^ interactions constitute a bifurcated association generating an asymmetric *R*^2^_1_(5) ring motif. The one-dimensional chain structures extend across [101] (Fig. 2) while the crystal structure is further stabilized by weak C—H···O hydrogen bonds and by π–π stacking interactions between inversion-related benzene rings [ring centroid–centroid distance = 3.7965 (10)Å] (Fig. 3), giving an overall three-dimensional structure.

### S2. Experimental

A mixture of ethyl acetoacetate (0.13 ml, 1 mmol), 3,4,5-trimethoxybenzaldehyde (0.196 g, 1 mmol), and urea (0.18 g, 3 mmol) in ethanol (5 ml) was heated under reflux in the presence of cerium chloride heptahydrate (25%) for 1 h (monitored by TLC). After the completion of the reaction, the reaction mixture was cooled to room temperature and poured onto crushed ice and stirred for 5–10 min. The solid was separated and filtered under suction, washed with ice-cold water (50 ml) and then recrystallized from hot ethanol to afford the pure product [m.p. 445 K; yield 96%].

### S3. Refinement

H atoms were placed in geometrically idealized positions and constrained to ride on their parent atoms with C—H distances fixed in the range 0.93–0.97 Å and N—H = 0.86 Å with *U*_iso_(H) = 1.5*U*_eq_(CH_3_) and 1.2*U*_eq_(CH_2_, CH, NH).

### Crystal data

**Table:**

C_17_H_22_N_2_O_6_	*Z* = 2
*M**_r_* = 350.37	*F*(000) = 372
Triclinic, *P*1	*D*_x_ = 1.313 Mg m^−^^3^
Hall symbol: -P 1	Melting point: 445 K
*a* = 10.1447 (3) Å	Mo *K*α radiation, λ = 0.71073 Å
*b* = 10.1919 (2) Å	Cell parameters from 3659 reflections
*c* = 10.8724 (2) Å	θ = 1.0–26.5°
α = 117.882 (1)°	µ = 0.10 mm^−^^1^
β = 101.371 (1)°	*T* = 293 K
γ = 105.498 (1)°	Block, colourless
*V* = 886.40 (4) Å^3^	0.20 × 0.15 × 0.10 mm

### Data collection

**Table:**

Bruker Kappa APEXII CCD diffractometer	3659 independent reflections
Radiation source: fine-focus sealed tube	3009 reflections with *I* > 2σ(*I*)
Graphite monochromator	*R*_int_ = 0.020
φ and ω scans	θ_max_ = 26.5°, θ_min_ = 2.3°
Absorption correction: multi-scan (*SADABS*; Bruker, 2008)	*h* = −12→12
*T*_min_ = 0.970, *T*_max_ = 0.995	*k* = −12→12
13060 measured reflections	*l* = −13→13

### Refinement

**Table:**

Refinement on *F*^2^	Secondary atom site location: difference Fourier map
Least-squares matrix: full	Hydrogen site location: inferred from neighbouring sites
*R*[*F*^2^ > 2σ(*F*^2^)] = 0.050	H-atom parameters constrained
*wR*(*F*^2^) = 0.154	*w* = 1/[σ^2^(*F*_o_^2^) + (0.0769*P*)^2^ + 0.2863*P*] where *P* = (*F*_o_^2^ + 2*F*_c_^2^)/3
*S* = 1.06	(Δ/σ)_max_ < 0.001
3659 reflections	Δρ_max_ = 0.60 e Å^−^^3^
228 parameters	Δρ_min_ = −0.30 e Å^−^^3^
1 restraint	Extinction correction: *SHELXL97* (Sheldrick, 2008), Fc^*^=kFc[1+0.001xFc^2^λ^3^/sin(2θ)]^-1/4^
Primary atom site location: structure-invariant direct methods	Extinction coefficient: 0.023 (4)

### Special details

**Table:**

Geometry. All e.s.d.'s (except the e.s.d. in the dihedral angle between two l.s. planes) are estimated using the full covariance matrix. The cell e.s.d.'s are taken into account individually in the estimation of e.s.d.'s in distances, angles and torsion angles; correlations between e.s.d.'s in cell parameters are only used when they are defined by crystal symmetry. An approximate (isotropic) treatment of cell e.s.d.'s is used for estimating e.s.d.'s involving l.s. planes.
Refinement. Refinement of *F*^2^ against ALL reflections. The weighted *R*-factor *wR* and goodness of fit *S* are based on *F*^2^, conventional *R*-factors *R* are based on *F*, with *F* set to zero for negative *F*^2^. The threshold expression of *F*^2^ > σ(*F*^2^) is used only for calculating *R*-factors(gt) *etc*. and is not relevant to the choice of reflections for refinement. *R*-factors based on *F*^2^ are statistically about twice as large as those based on *F*, and *R*- factors based on ALL data will be even larger.

### Fractional atomic coordinates and isotropic or equivalent isotropic displacement parameters (Å^2^)

**Table:**

	*x*	*y*	*z*	*U*_iso_*/*U*_eq_	
C1	0.37112 (18)	0.3299 (2)	0.72474 (17)	0.0394 (4)	
C2	0.44867 (19)	0.2873 (2)	0.80886 (18)	0.0410 (4)	
H2	0.4009	0.1946	0.8073	0.049*	
C3	0.59827 (19)	0.3837 (2)	0.89570 (18)	0.0415 (4)	
C4	0.66902 (18)	0.5242 (2)	0.90186 (18)	0.0429 (4)	
C5	0.5914 (2)	0.5645 (2)	0.8146 (2)	0.0477 (4)	
C6	0.4429 (2)	0.4661 (2)	0.7249 (2)	0.0466 (4)	
H6	0.3916	0.4918	0.6647	0.056*	
C7	0.6280 (3)	0.2077 (3)	0.9753 (3)	0.0698 (6)	
H7A	0.7016	0.2040	1.0427	0.105*	
H7B	0.5992	0.1174	0.8743	0.105*	
H7C	0.5433	0.2011	1.0028	0.105*	
C8	0.9201 (2)	0.6153 (3)	0.9346 (2)	0.0652 (6)	
H8A	1.0172	0.6907	1.0103	0.098*	
H8B	0.9011	0.6404	0.8599	0.098*	
H8C	0.9141	0.5062	0.8882	0.098*	
C9	0.5979 (3)	0.7486 (3)	0.7382 (4)	0.0828 (8)	
H9A	0.6640	0.8501	0.7591	0.124*	
H9B	0.5124	0.7596	0.7590	0.124*	
H9C	0.5679	0.6647	0.6343	0.124*	
C10	0.0111 (2)	0.1030 (3)	0.2264 (2)	0.0599 (5)	
H10A	−0.0726	0.1254	0.2006	0.090*	
H10B	−0.0203	−0.0123	0.1806	0.090*	
H10C	0.0824	0.1408	0.1908	0.090*	
C11	0.07976 (19)	0.1893 (2)	0.39399 (19)	0.0445 (4)	
C12	0.15090 (18)	0.1416 (2)	0.47179 (19)	0.0425 (4)	
C13	0.20307 (18)	0.2357 (2)	0.64192 (18)	0.0410 (4)	
H13	0.1699	0.1573	0.6694	0.049*	
C14	0.0864 (2)	0.4142 (2)	0.62100 (19)	0.0455 (4)	
C15	0.1840 (2)	−0.0008 (2)	0.3951 (2)	0.0496 (4)	
C16	0.2722 (3)	−0.1808 (3)	0.4190 (3)	0.0833 (8)	
H16A	0.3437	−0.1572	0.3764	0.100*	
H16B	0.1855	−0.2781	0.3388	0.100*	
C17	0.3362 (5)	−0.2078 (4)	0.5344 (4)	0.1191 (13)	
H17A	0.3634	−0.2977	0.4898	0.179*	
H17B	0.2647	−0.2321	0.5754	0.179*	
H17C	0.4223	−0.1113	0.6131	0.179*	
N1	0.06044 (18)	0.33131 (19)	0.47041 (16)	0.0507 (4)	
H1N	0.0304	0.3699	0.4205	0.061*	
N2	0.13307 (16)	0.34918 (19)	0.69333 (16)	0.0447 (4)	
H2N	0.1210	0.3761	0.7766	0.054*	
O1	0.1753 (2)	−0.07004 (19)	0.26618 (17)	0.0744 (5)	
O2	0.23206 (16)	−0.04531 (16)	0.48721 (16)	0.0609 (4)	
O3	0.68614 (15)	0.35473 (16)	0.98399 (14)	0.0559 (4)	
O4	0.81300 (14)	0.62806 (16)	1.00213 (14)	0.0542 (4)	
O5	0.67016 (17)	0.70646 (19)	0.8294 (2)	0.0717 (5)	
O6	0.06186 (17)	0.53697 (18)	0.68139 (15)	0.0598 (4)	

### Atomic displacement parameters (Å^2^)

**Table:**

	*U*^11^	*U*^22^	*U*^33^	*U*^12^	*U*^13^	*U*^23^
C1	0.0405 (8)	0.0409 (8)	0.0349 (8)	0.0214 (7)	0.0128 (7)	0.0178 (7)
C2	0.0455 (9)	0.0391 (8)	0.0371 (8)	0.0222 (7)	0.0126 (7)	0.0189 (7)
C3	0.0465 (9)	0.0450 (9)	0.0316 (8)	0.0272 (7)	0.0116 (7)	0.0170 (7)
C4	0.0390 (8)	0.0457 (9)	0.0338 (8)	0.0192 (7)	0.0105 (6)	0.0153 (7)
C5	0.0468 (9)	0.0465 (9)	0.0505 (10)	0.0197 (8)	0.0179 (8)	0.0273 (8)
C6	0.0450 (9)	0.0532 (10)	0.0487 (10)	0.0246 (8)	0.0138 (8)	0.0324 (8)
C7	0.0782 (15)	0.0662 (13)	0.0680 (13)	0.0347 (12)	0.0081 (11)	0.0440 (11)
C8	0.0440 (10)	0.0744 (14)	0.0590 (12)	0.0240 (10)	0.0159 (9)	0.0256 (11)
C9	0.0771 (16)	0.0827 (17)	0.117 (2)	0.0329 (14)	0.0329 (15)	0.0762 (17)
C10	0.0654 (12)	0.0652 (12)	0.0397 (10)	0.0334 (10)	0.0109 (9)	0.0223 (9)
C11	0.0431 (9)	0.0451 (9)	0.0386 (9)	0.0197 (7)	0.0121 (7)	0.0190 (7)
C12	0.0385 (8)	0.0399 (8)	0.0397 (9)	0.0160 (7)	0.0099 (7)	0.0174 (7)
C13	0.0402 (8)	0.0438 (9)	0.0409 (9)	0.0207 (7)	0.0127 (7)	0.0240 (7)
C14	0.0454 (9)	0.0535 (10)	0.0405 (9)	0.0290 (8)	0.0156 (7)	0.0237 (8)
C15	0.0433 (9)	0.0399 (9)	0.0471 (10)	0.0154 (7)	0.0070 (7)	0.0157 (8)
C16	0.0882 (17)	0.0513 (12)	0.0835 (17)	0.0417 (12)	0.0098 (13)	0.0202 (12)
C17	0.142 (3)	0.094 (2)	0.107 (2)	0.079 (2)	0.012 (2)	0.0433 (19)
N1	0.0662 (10)	0.0574 (9)	0.0382 (8)	0.0390 (8)	0.0178 (7)	0.0269 (7)
N2	0.0461 (8)	0.0586 (9)	0.0364 (7)	0.0312 (7)	0.0170 (6)	0.0257 (7)
O1	0.0979 (12)	0.0630 (9)	0.0499 (9)	0.0456 (9)	0.0243 (8)	0.0172 (7)
O2	0.0668 (9)	0.0449 (7)	0.0571 (8)	0.0302 (7)	0.0086 (7)	0.0203 (6)
O3	0.0544 (8)	0.0581 (8)	0.0487 (7)	0.0271 (6)	0.0034 (6)	0.0289 (6)
O4	0.0419 (7)	0.0570 (8)	0.0397 (7)	0.0149 (6)	0.0088 (5)	0.0152 (6)
O5	0.0554 (8)	0.0653 (9)	0.0974 (12)	0.0163 (7)	0.0160 (8)	0.0564 (9)
O6	0.0778 (10)	0.0686 (9)	0.0446 (7)	0.0525 (8)	0.0231 (7)	0.0277 (7)

### Geometric parameters (Å, º)

**Table:**

C1—C2	1.384 (2)	C10—C11	1.501 (2)
C1—C6	1.384 (2)	C10—H10A	0.9600
C1—C13	1.533 (2)	C10—H10B	0.9600
C2—C3	1.389 (2)	C10—H10C	0.9600
C2—H2	0.9300	C11—C12	1.344 (2)
C3—O3	1.368 (2)	C11—N1	1.383 (2)
C3—C4	1.383 (3)	C12—C15	1.468 (2)
C4—O4	1.382 (2)	C12—C13	1.518 (2)
C4—C5	1.390 (3)	C13—N2	1.464 (2)
C5—O5	1.368 (2)	C13—H13	0.9800
C5—C6	1.387 (3)	C14—O6	1.233 (2)
C6—H6	0.9300	C14—N2	1.338 (2)
C7—O3	1.406 (3)	C14—N1	1.371 (2)
C7—H7A	0.9600	C15—O1	1.210 (2)
C7—H7B	0.9600	C15—O2	1.340 (2)
C7—H7C	0.9600	C16—O2	1.444 (3)
C8—O4	1.428 (2)	C16—C17	1.472 (3)
C8—H8A	0.9600	C16—H16A	0.9700
C8—H8B	0.9600	C16—H16B	0.9700
C8—H8C	0.9600	C17—H17A	0.9600
C9—O5	1.410 (3)	C17—H17B	0.9600
C9—H9A	0.9600	C17—H17C	0.9600
C9—H9B	0.9600	N1—H1N	0.8600
C9—H9C	0.9600	N2—H2N	0.8600
			
C2—C1—C6	120.13 (15)	H10B—C10—H10C	109.5
C2—C1—C13	120.26 (15)	C12—C11—N1	119.53 (15)
C6—C1—C13	119.40 (15)	C12—C11—C10	127.51 (17)
C1—C2—C3	119.70 (16)	N1—C11—C10	112.94 (15)
C1—C2—H2	120.1	C11—C12—C15	120.97 (16)
C3—C2—H2	120.2	C11—C12—C13	121.07 (15)
O3—C3—C4	114.49 (15)	C15—C12—C13	117.94 (15)
O3—C3—C2	125.05 (16)	N2—C13—C12	109.34 (13)
C4—C3—C2	120.42 (15)	N2—C13—C1	109.29 (14)
O4—C4—C3	119.43 (15)	C12—C13—C1	114.52 (14)
O4—C4—C5	120.85 (16)	N2—C13—H13	107.8
C3—C4—C5	119.58 (16)	C12—C13—H13	107.8
O5—C5—C6	124.58 (17)	C1—C13—H13	107.8
O5—C5—C4	115.35 (16)	O6—C14—N2	123.56 (16)
C6—C5—C4	120.03 (17)	O6—C14—N1	120.72 (16)
C1—C6—C5	120.03 (16)	N2—C14—N1	115.66 (15)
C1—C6—H6	120.0	O1—C15—O2	122.09 (18)
C5—C6—H6	120.0	O1—C15—C12	125.98 (18)
O3—C7—H7A	109.5	O2—C15—C12	111.89 (16)
O3—C7—H7B	109.5	O2—C16—C17	109.0 (2)
H7A—C7—H7B	109.5	O2—C16—H16A	109.9
O3—C7—H7C	109.5	C17—C16—H16A	109.9
H7A—C7—H7C	109.5	O2—C16—H16B	109.9
H7B—C7—H7C	109.5	C17—C16—H16B	109.9
O4—C8—H8A	109.5	H16A—C16—H16B	108.3
O4—C8—H8B	109.5	C16—C17—H17A	109.5
H8A—C8—H8B	109.5	C16—C17—H17B	109.5
O4—C8—H8C	109.5	H17A—C17—H17B	109.5
H8A—C8—H8C	109.5	C16—C17—H17C	109.5
H8B—C8—H8C	109.5	H17A—C17—H17C	109.5
O5—C9—H9A	109.5	H17B—C17—H17C	109.5
O5—C9—H9B	109.5	C14—N1—C11	123.90 (15)
H9A—C9—H9B	109.5	C14—N1—H1N	118.1
O5—C9—H9C	109.5	C11—N1—H1N	118.1
H9A—C9—H9C	109.5	C14—N2—C13	125.19 (14)
H9B—C9—H9C	109.5	C14—N2—H2N	117.4
C11—C10—H10A	109.5	C13—N2—H2N	117.4
C11—C10—H10B	109.5	C15—O2—C16	114.13 (17)
H10A—C10—H10B	109.5	C3—O3—C7	118.70 (15)
C11—C10—H10C	109.5	C4—O4—C8	113.93 (14)
H10A—C10—H10C	109.5	C5—O5—C9	117.35 (17)
			
C6—C1—C2—C3	1.3 (2)	C6—C1—C13—N2	−52.6 (2)
C13—C1—C2—C3	−173.41 (15)	C2—C1—C13—C12	−114.87 (17)
C1—C2—C3—O3	179.57 (16)	C6—C1—C13—C12	70.4 (2)
C1—C2—C3—C4	1.8 (2)	C11—C12—C15—O1	12.9 (3)
O3—C3—C4—O4	−5.5 (2)	C13—C12—C15—O1	−165.48 (19)
C2—C3—C4—O4	172.46 (15)	C11—C12—C15—O2	−169.40 (16)
O3—C3—C4—C5	178.81 (15)	C13—C12—C15—O2	12.2 (2)
C2—C3—C4—C5	−3.2 (3)	O6—C14—N1—C11	−177.32 (18)
O4—C4—C5—O5	3.7 (3)	N2—C14—N1—C11	0.0 (3)
C3—C4—C5—O5	179.34 (16)	C12—C11—N1—C14	−11.8 (3)
O4—C4—C5—C6	−174.07 (16)	C10—C11—N1—C14	166.62 (18)
C3—C4—C5—C6	1.6 (3)	O6—C14—N2—C13	−161.54 (18)
C2—C1—C6—C5	−2.9 (3)	N1—C14—N2—C13	21.2 (3)
C13—C1—C6—C5	171.79 (16)	C12—C13—N2—C14	−27.1 (2)
O5—C5—C6—C1	−176.06 (17)	C1—C13—N2—C14	98.96 (19)
C4—C5—C6—C1	1.5 (3)	O1—C15—O2—C16	0.3 (3)
N1—C11—C12—C15	−174.94 (16)	C12—C15—O2—C16	−177.52 (18)
C10—C11—C12—C15	6.9 (3)	C17—C16—O2—C15	174.6 (2)
N1—C11—C12—C13	3.4 (3)	C4—C3—O3—C7	−174.43 (18)
C10—C11—C12—C13	−174.76 (18)	C2—C3—O3—C7	7.7 (3)
C11—C12—C13—N2	13.6 (2)	C3—C4—O4—C8	102.7 (2)
C15—C12—C13—N2	−167.97 (15)	C5—C4—O4—C8	−81.7 (2)
C11—C12—C13—C1	−109.38 (18)	C6—C5—O5—C9	−5.1 (3)
C15—C12—C13—C1	69.0 (2)	C4—C5—O5—C9	177.3 (2)
C2—C1—C13—N2	122.09 (16)		

### Hydrogen-bond geometry (Å, º)

**Table:**

*D*—H···*A*	*D*—H	H···*A*	*D*···*A*	*D*—H···*A*
N1—H1*N*···O6^i^	0.86	2.01	2.867 (2)	171
N2—H2*N*···O3^ii^	0.86	2.56	3.0629 (19)	118
N2—H2*N*···O4^ii^	0.86	2.39	3.1331 (19)	145
C8—H8*A*···O1^iii^	0.96	2.46	3.325 (3)	149
C9—H9*A*···O1^iv^	0.96	2.56	3.491 (3)	163

Symmetry codes: (i) −*x*, −*y*+1, −*z*+1; (ii) −*x*+1, −*y*+1, −*z*+2; (iii) *x*+1, *y*+1, *z*+1; (iv) −*x*+1, −*y*+1, −*z*+1.
