# Supplementary figures and images for: Crystal structure of ethyl 6-methyl-2-oxo-4-(3,4,5-tri­meth­oxy­phen­yl)-1,2,3,4-tetra­hydro­pyrimidine-5-carboxyl­ate
Source: Acta Crystallogr E Crystallogr Commun. 2015 Feb 25;71(Pt 3):o206–7. doi: 10.1107/S2056989015003576 (PMC4350754; doi:10.1107/S2056989015003576)

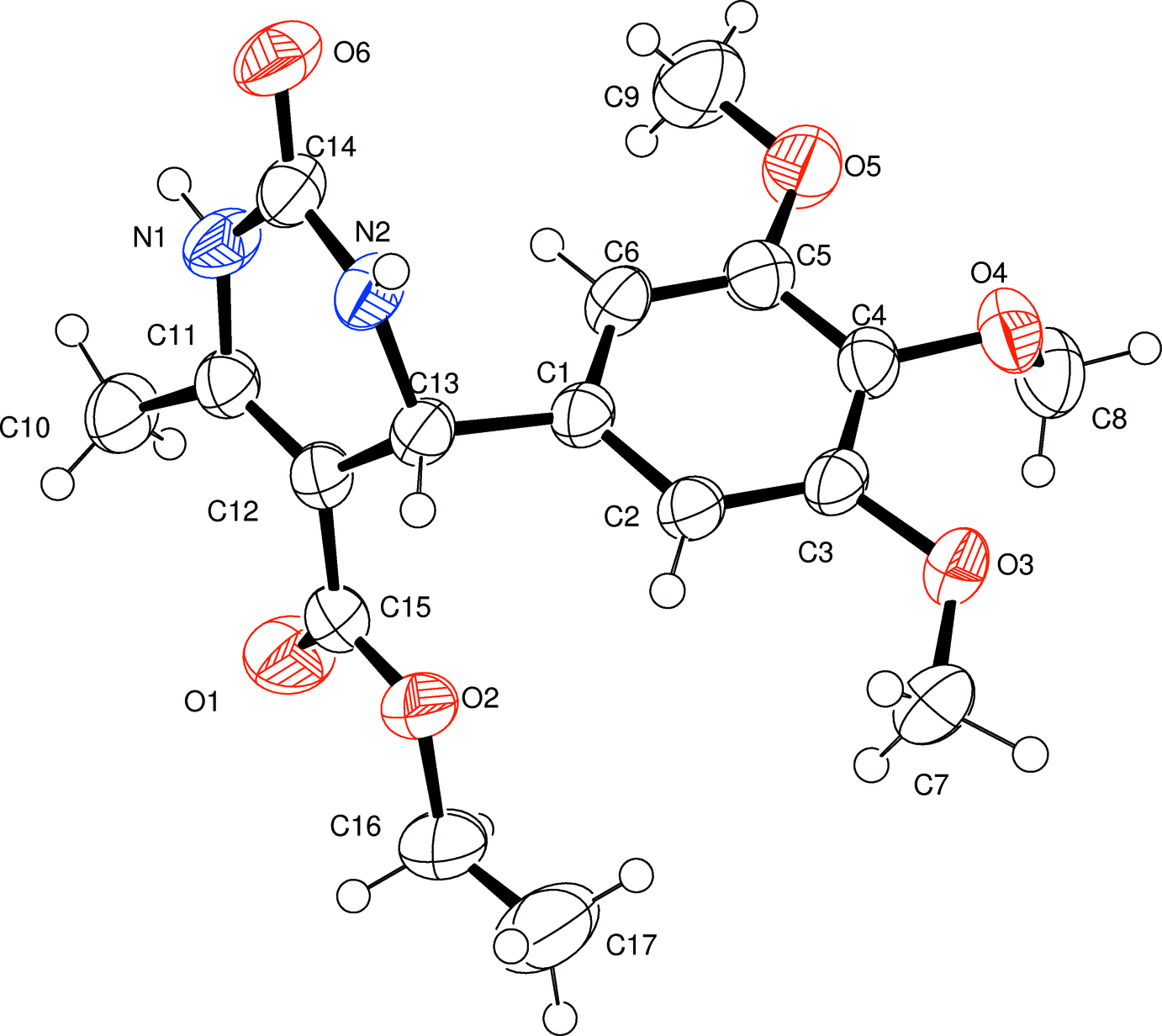

Supplement: Supplementary file 4 [file e-71-0o206-fig1.tif]

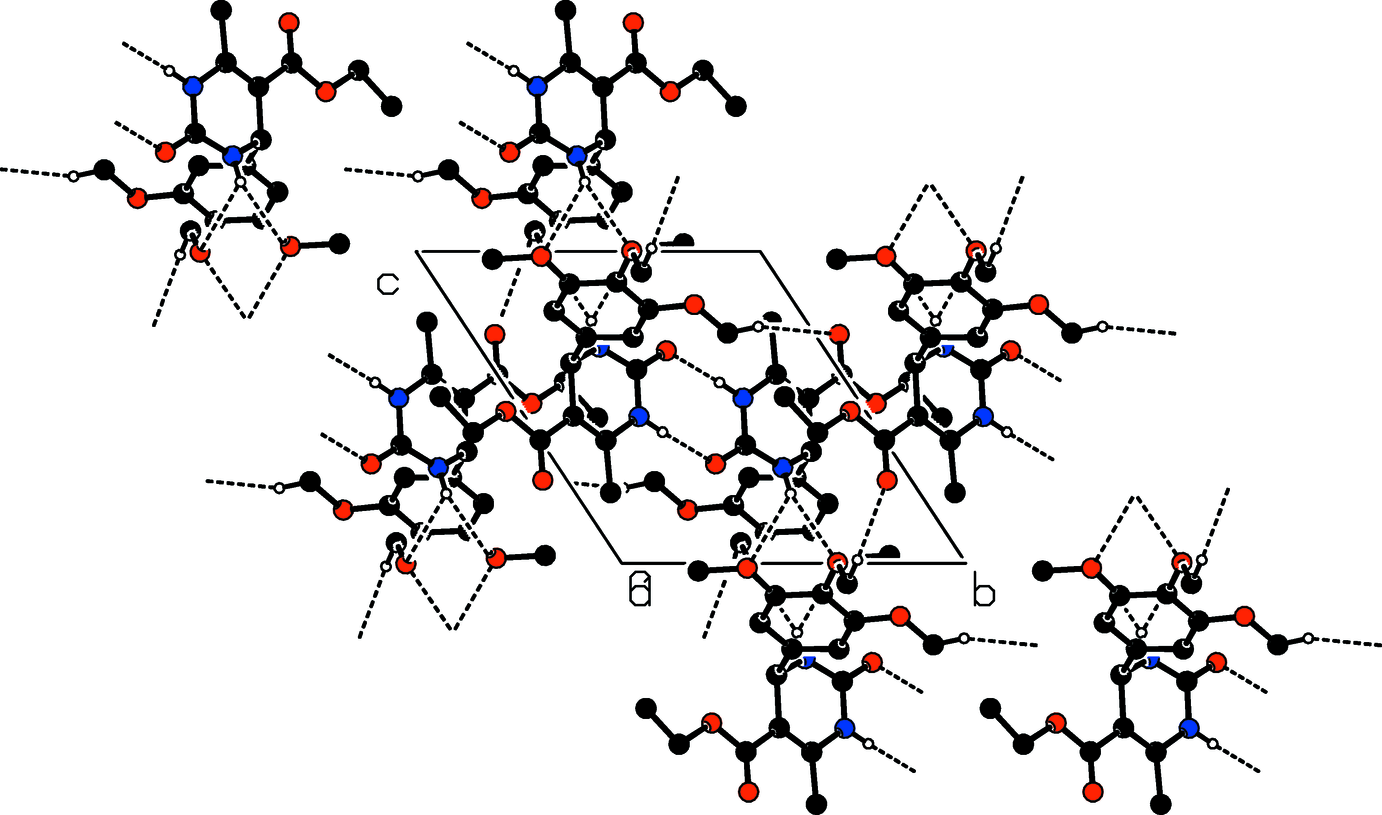

Supplement: Supplementary file 5 [file e-71-0o206-fig2.tif]

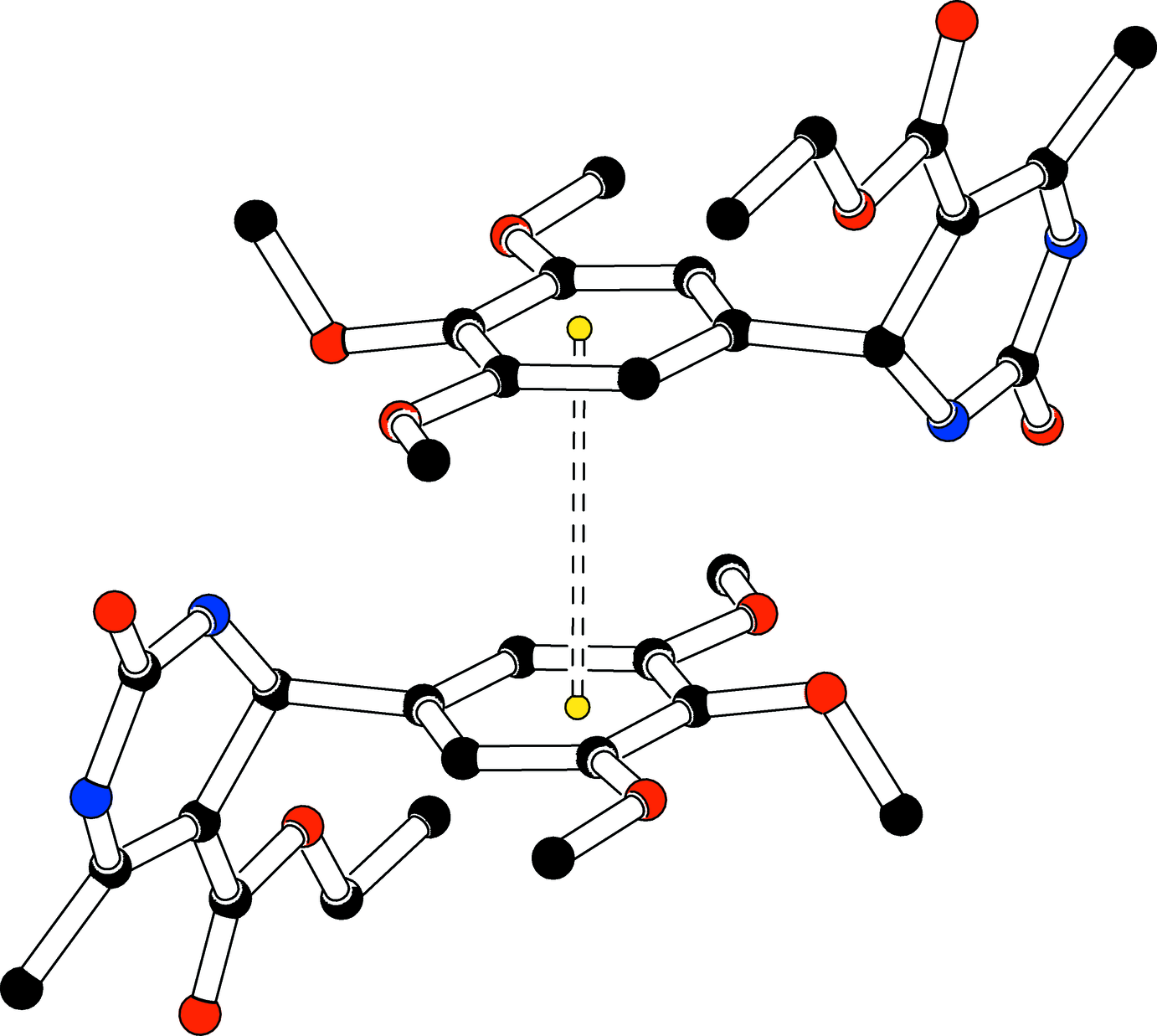

Supplement: Supplementary file 6 [file e-71-0o206-fig3.tif]
